# Degradability, rumen fermentation, and rumen microbiota of livestock rations containing different levels of *Azolla pinnata*

**DOI:** 10.3934/microbiol.2025028

**Published:** 2025-08-01

**Authors:** Ahmed. F.A. Abd-Elgwad, Salah Abo Bakr, Ebrahim A. Sabra, Eman A. Elwakeel, Mahmoud. M. Khorshed, Hamdy. M. Metwally, Alaa Emara Rabee

**Affiliations:** 1 Animal and Poultry Nutrition Department, Desert Research Center, Cairo, Egypt; 2 Genetic Engineering and Biotechnology Research Institute, University of Sadat City, Sadat City, Egypt; 3 Animal and Fish Production Department, Faculty of Agriculture, Alexandria University, Alexandria; 4 Animal Production Department, Faculty of Agriculture, Ain-Shams University, Cairo, Egypt

**Keywords:** *Azolla pinnata*, nutrients degradability, rumen fermentation, microbiota

## Abstract

The scarcity of animal feeding resources has been driving the use of sustainable alternatives such as *Azolla*. This study evaluated the effect of replacing concentrate feed mixture (CFM) with dried *Azolla* (DAZ) on the *in vitro* digestibility of rations, rumen fermentations, gas production, and rumen microbiota. The basal diet consisted of Berseem hay and CFM (50:50), and six rations were used, in which DAZ replaced the CFM at 0% (control), 10% (T1), 20% (T2), 30% (T3), 40% (T4), and 50% (T5). Group T1 showed higher degradability of dry matter (53.13%), organic matter (62.47%), neutral detergent fiber (30.79%), and acid detergent fiber (24.72%). The same group (T1) revealed the highest propionate and lowest methane production (p < 0.05). Principal coordinate analysis (PCoA) revealed that rumen microbial communities were affected by DAZ level. Microbial communities were dominated by the phylum Bacteroidota, which was higher in group T1, and the phylum Firmicutes, which was higher in group T2. The dominant bacterial genera were *Prevotella*, *Rikenellaceae RC9 gut group*, *Streptococcus*, and *Christensenellaceae R-7 group*, which were affected by DAZ level. Dried *Azolla* can be used up to 20% of CFM in ruminant rations without negative consequences on rumen fermentation.

## 1. Introduction

The low availability of animal feeds is the major constraint in livestock production in developing countries. Therefore, unconventional feed resources are being incorporated into animal rations to increase animal products and meet the demand of the increasing world population [1]–[3]. Moreover, using unconventional feed resources such as *Azolla* could ensure profitable animal production due to the low cost and production under local conditions [4],[5]. *Azolla pinnata* is a floating fern that commonly grows on the water surface in tropical regions. Azolla requires minimal land area compared to conventional fodder crops, making it an economic feed resource [2],[4].

*Azolla* is a rich source of proteins, essential amino acids, essential fatty acids, vitamins, and minerals [2],[3]. Additionally, it has a low lignin content, which improves its rumen digestibility. Therefore, *Azolla* has attracted attention as a partial replacement to concentrated feed mixtures [3]. In addition, *Azolla* contains different phytochemical substances such as phenols, flavonoids, and saponins that can act as antioxidants, anti-inflammatories, immune modulators, and antimicrobials, which improve animal health and modify the rumen microbiota [3].

Rumen microbiota is dominated by bacteria that perform most of the rumen fermentation of ingested feed due to the diversity of bacterial species, which ferment several dietary substrates [6]. Rumen methanogens produce methane (CH_4_) using hydrogen (H_2_) and carbon dioxide (CO_2_), besides other substrates such as acetate, formate, and methanol. CH_4_ represents a loss of up to 12% of the gross energy feed intake of the host animal, and it is considered one of the main greenhouse gases that causes global warming [7]. Rumen bacteria and archaea showed different levels of sensitivity against dietary phytochemicals; thus, *Azolla* can impact rumen microbial fermentation [7],[8].

No information is available on the effect of *Azolla* on the rumen microbiota. However, the inclusion of phytogenic compounds in goats' diets improved volatile fatty acids (VFA), enriched fiber-degrading bacteria such as *Prevotella* and *Rikenellaceae RC9 gut group*, and reduced methane production and main rumen methanogens [7]. Moreover, the inclusion of *Azolla* into straw-based rations improved *in vivo* and *in vitro* digestibility [1],[9]. Furthermore, rumen ammonia was increased by increasing *Azolla* level, while higher VFA were observed when *Azolla* replaced 10% of the CFM [4]. Furthermore, inclusion of *Azolla* up to 20% of the CFM of lactating goats improved the digestibility, milk yield, and rumen fermentation [3]. On the other hand, Kavya, et al. [10] concluded that *Azolla* can only serve as a good source of protein, amino acids, and minerals without beneficial effects on the digestibility or energy density of rations. Subsequently, there is no agreement on the suitable level of *Azolla* in animal diets; thus, it is necessary to assign suitable levels of *Azolla* supplementation in animal diets and to evaluate the effect of its supplementation level on rumen microbiota. Therefore, this study aimed to investigate the digestibility of nutrients, rumen fermentation, and rumen microbiota associated with rations with different levels of *Azolla*.

## 2. Materials and methods

### 2.1. Ethics

This study was carried out at Maryout Research Station, Desert Research Center, Alexandria, Egypt. This study does not include any clinical trials on animals, and the study, including rumen collection from animals, was conducted under the guidelines and the permissions of the Animal Care and Use Committee in the Division of Animal and Poultry Production, Desert Research Center, Egypt (reference number: AP-AZ-2024). All methods and protocols in this study comply with the ARRIVE 2.0 guidelines. The animals were used based on a required consent from the administration of Maryout Research Station and the Animal and Poultry Production Division. The experiment does not include animal anesthesia or euthanasia, and the animals were released to the experimental sheep herd at the end of the experiment.

### 2.2. Preparation of *Azolla*

*Azolla* (*Azolla Pinnata*) was grown in Maryout Research Station, Desert Research Center, Alexandria, Egypt. The Azolla was grown in 2 × 3 m plastic-lined ponds with 10–15 cm of soft soil and 30 cm of water over the plastic sheet. The pond was fertilized with 500 g of superphosphate and 15 kg of sheep feces fermented in 40 L of water that was distributed throughout the pond and repeated every 15 days. Three kilograms of fresh *Azolla pinnata* was used in the *Azolla* cultivation. *Azolla* was harvested after 10 days when a dense mat had formed. On the harvesting day, *Azolla* was collected, washed with clean water, and left for the water to drain. Then, *Azolla* was weighed to determine the fresh quantity (8 kg fresh *Azolla*/m^2^) and sun-dried for 5 days to determine humidity (90%). Sundried *Azolla* was further oven-dried at 50 °C for 48 h and ground into 0.3 mm pieces to be mixed with the ration's ingredients.

### 2.3. In vitro incubation of experimental rations

The *in vitro* gas production technique of Menke, et al. [11] was used to estimate the effect of *Azolla* inclusion level in animal rations on the dry matter degradability (DMD), organic matter degradability (OMD), neutral detergent fiber degradability (NDFD), gas production [total gas, carbon dioxide (CO_2_), and methane (CH_4_)], rumen fermentation parameters (pH, ammonia, VFA), and associated bacterial community. Six rations were used in this study, as shown in Table 1. The control diet consisted of 50% Berseem hay and 50% concentrate feed mixture (CFM); in T1, 10% of CFM was replaced by dried *Azolla*; in T2, 20% of CFM was replaced by dried *Azolla*; in T3, 30% of CFM was replaced by dried *Azolla*; in T4, 40% of CFM was replaced by dried *Azolla*; and in T5, 50% of CFM was replaced by dried *Azolla*.

The CFM consisted of 60% corn, 11.5% soybean meal, 25% Wheat bran, 1.5% limestone, 1.5% salt, and 0.5% premix. The chemical compositions of CFM, Berseem hay, and the examined rations are presented in Table 1. Rumen content was collected via a cannula of three ruminally cannulated rams fed Berseem hay. Rumen contents of the rams were squeezed through a four-layer cheesecloth, and the liquids were pooled in one jar, which was incubated in a water bath at 39 °C saturated with CO_2_ until inoculation. The incubation medium was prepared according to Menke, et al. [11].

Five serum bottles were used for each diet (n = 5), accompanied by five blank bottles (without substrates). The tested rations (400 mg) were added to the 125 mL serum bottles. Each serum bottle was filled with 40 mL of a 1:3 (v/v) mixture of rumen fluids and buffer solution. The serum bottles were sealed and incubated at 39 °C for 24 h. The cumulated gas production was monitored and recorded using a graduated syringe. Values were corrected for blank value, and gas yield was expressed as mL per 400 mg of DM per 24 h. Both CH_4_ and CO_2_ productions were recorded at 24 h of incubation using a Gas-Pro detector (Gas Analyzer CROWCON, Model Tetra3, Abingdon, UK).

**Table 1.** Chemical composition of the experimental rations and their ingredients (%) on a dry matter basis.

Items	Control	T1	T2	T3	T4	T5	*Azolla*	CFM	Berseem Hay
Chemical composition (%)									
DM	92.22	92.21	92.20	92.17	92.18	92.16	92.90	93.00	91.42
OM	91.80	91.00	90.20	89.33	88.60	87.70	79.00	95.00	88.72
EE	4.10	4.15	4.20	4.25	4.30	4.30	4.70	4.00	4.18
CP	12.20	12.47	12.66	12.90	13.12	13.33	17.18	13.00	12.00
NDF	47.88	48.50	49.05	49.61	50.22	50.82	53.00	41.60	53.95
ADF	20.60	21.81	23.11	24.35	25.60	26.82	31.63	6.92	34.00
Ash	8.20	9.00	9.80	10.67	11.40	12.30	21.00	5.00	11.28
Percentage (%) of ingredients in experimental rations									
CFM	50%	45%	40%	35%	30%	25%			
Dried *Azolla*	0%	5%	10%	15%	20%	25%			
Berseem hay	50%	50%	50%	50%	50%	50%			
Total	100	100	100	100	100	100			
Percentage (%) of *Azolla* in CFM									
Dried *Azolla*	0%	10%	20%	30%	40%	50%			

DM = Dry matter; OM = organic matter; CP = crude protein; EE = ether extract; NDF = neutral detergent fiber; ADF = acid detergent fiber; CFM = concentrate feed mixture.

### 2.4. Dry matter degradation and fermentation parameters of experimental rations

After 24 h, the contents of the bottles were vortexed vigorously for 10 min to dissociate the solid-attached microorganisms and then filtered using nylon bags with 25-micron porosity (ANKOM-USA). The liquid was transferred to a 50 mL tube. The solid content was rinsed using distilled water, oven-dried at 60 °C for 48 h, and weighed to determine DMD. A sample of 2 mL of liquid from each bottle was transferred to a 2 mL tube and frozen immediately for DNA extraction to identify the bacterial community. The pH of *in vitro* fermentation liquids was measured in the rest of the liquid samples using a pH meter. The liquid samples were centrifuged at 13,000 rpm for 15 min to determine the concentration of ammonia and VFAs. Ammonia concentration was determined in the liquids using an ammonia assay kit (Biodiagnostic, Cairo, Egypt). VFAs were determined using a capillary column (TR-FFAP 30 m × 0.53 mm D × 0.5 µm) in a Thermo Scientific TRACE 1300 gas chromatography system (Thermo Scientific, Massachusetts, United States) as described in Rabee, et al. [7].

### 2.5. Chemical analyses of experimental rations

The residues of undigested materials, dried *Azolla*, and rations were dried, ground, and analyzed according to AOAC [12] to determine dry matter (DM), organic matter (OM), crude protein (CP), and ether extract (EE). Additionally, neutral detergent fiber (NDF) and acid detergent fiber (ADF) were measured according to Van Soest, et al. [13] using an ANKOM 200 Fiber Analyzer (ANKOM Technology, New York, United States). Total flavonoids, total phenols, and total tannins were measured in *Azolla*. Total phenol was measured using Folin–Ciocalteu [14]. Total tannins were extracted by boiling in water and measured according to Balbaa [15]. Total flavonoids were extracted with petroleum ether and 95% ethanol and measured according to Karawaya and Aboutabl [16]. Phenolic compounds were determined using high-performance liquid chromatography (HPLC) (Thermo Scientific, Massachusetts, United States) using a reversed-phase C18 column. The mobile phases consisted of water (A) and 0.05% trifluoroacetic acid in acetonitrile (B) at a flow rate of 0.9 mL/min [17].

### 2.6. Fatty acid profile of Azolla

To determine fatty acids in *Azolla*, lipids were extracted by the biphasic 2:1 chloroform/methanol (v/v) Folch extraction method. Fatty acid methyl esters (FAME) were produced by an alkali-catalyzed reaction between fats and methanol in the presence of 2 M potassium hydroxide and injected in hexane. Fatty acids were measured using a gas chromatography system, model 7890B (Thermo Scientific, Massachusetts, United States). Separation was achieved using a Zebron ZB-FAME column (60 m × 0.25 mm internal diameter × 0.25 µm film thickness). Analyses were carried out using hydrogen as the carrier gas at a flow rate of 1.8 mL/min at a split 1:50 mode, injection volume of 1 µL, and the following temperature program: 100 °C for 3 min; rising at 2.5 °C/min to 240 °C, and held for 10 min. The injector and detector (FID) were held at 250 °C and 285 °C, respectively.

### 2.7. Analysis of the microbial community associated with in vitro fermented rations

#### 2.7.1. DNA extraction and PCR amplification

500 µL of every fermentation liquid sample was centrifuged at 13,000 rpm for 15 min, and the remaining pellets were used for DNA extraction using a QIAamp DNA Stool Mini kit (Qiagen, Hilden, Germany) according to the manufacturer's instructions. The quality and quantity of DNA were determined by gel electrophoresis and a Nanodrop spectrophotometer 2000 (Thermo Scientific, Massachusetts, United States). DNA extraction was repeated in samples with degraded DNA or DNA concentration lower than 10 ng/µL. Then, the bacterial V4 regions on the 16S rDNA genes were amplified using primers 515F and 926R. PCR amplification was performed under the following cycling conditions: 94 °C for 3 min; 35 cycles of 94 °C for 45 s, 50 °C for 60 s, and 72 °C for 90 s; and 72 °C for 10 min. PCR amplicons were purified and sequenced using the Illumina MiSeq system (Illumina, California, United States).

#### 2.7.2. Bioinformatics analysis

The analyses of Illumine paired-end (PE) raw sequence reads were conducted in R (version 3.5.2) using the DADA2 pipeline (version 1.11.3) [18]. The generated fastq files were demultiplexed, and the qualities of forward and reverse reads were checked based on the quality scores. The clean sequence reads were denoised, dereplicated, and filtered for chimeras to generate Amplicon Sequence Variants (ASVs). The samples with a quality score > 30 were kept in the following analyses. Taxonomic assignment of ASVs was conducted using a combination of the functions assignTaxonomy and assignSpecies and compared using the latest version of the SILVA reference database (version 138). Alpha diversity metrics, observed ASVs, Chao1, Shannon, and Inverse Simpson were calculated, and Beta diversity was determined by principal coordinate analysis (PCoA) using Bray-Curtis dissimilarity and visualized using the phyloseq and ggplot packages. The raw sequence reads are available at https://www.ncbi.nlm.nih.gov/sra/PRJNA1141115.

#### 2.7.3. Copy number of rumen methanogens using the *mcrA* gene

Quantitative real-time PCR (qPCR) was carried out to quantify the total copy number of rumen methanogens using the copy number of the mcrA gene in 1 µL of isolated DNA. Standards were generated using dilutions of purified DNA from *Methanosphaera stadtmanae* and *Methanobrevibacter ruminantium* purchased from Deutsche Sammlung von Mikroorganismen und Zellkulturen (DSMZ), Germany. A dilution series of the standards, ranging from 10^1^ to 10^6^ copies of the *mcrA* gene, was applied. The qPCR was performed using the Applied Biosystems StepOne system (Applied Biosystems, Foster City, USA). The methanogens' specific primers qmcrA-R (5′-GBARGTCGWAWCCGTAGAATCC) and qmcrA-F (5′-TTCGGTGGATCDCARAGRGC) [19] were applied to amplify DNA samples and diluted standards. The 10-µL qPCR reaction consisted of 1 µL of genomic DNA, 1 µL of each primer, and 7 µL of SYBER Green qPCR master mix (iNtRON Biotechnology, Inc.). The qPCR cycle conditions were one cycle of 50 °C for 2 min and 95 °C for 2 min for initial denaturation, 40 cycles of 95 °C for 15 s, and 60 °C for 60 s. The total copy number of archaeal *mcrA* per 1 µL of DNA was measured using the linear relationship between the threshold amplification (Ct) and the logarithm of *mcrA* copy numbers of the standards.

### 2.8. Statistical analysis

The results of the relative abundances of bacterial phyla and genera were tested for normality and homogeneity using the Shapiro–Wilk test, and non-normal variables were arcsine transformed. The effect of *Azolla* supplementation level on the degradability of nutrients, rumen fermentation, and the relative abundances and diversity of rumen bacteria was examined by a Duncan test in a one-way ANOVA at p < 0.05. Principal component analysis (PCA) and Pearson correlation analysis (heatmap) were conducted based on data on the degradability of nutrients, rumen fermentation, and relative abundances and diversity of rumen bacteria. PCA was used to compare the clustering of samples due to the *Azolla* inclusion level, and the heatmap was used to show correlation relationships between nutrients' degradability, fermentation parameters, and relative abundances of microbial groups. The statistical analyses were performed using SPSS v. 20.0 software package [20] and PAST [21].

## 3. Results

### 3.1. Chemical composition of Azolla and experimental rations

The chemical composition of *Azolla*, Berseem hay, CFM, and experimental rations is presented in Table 1. In this study, *Azolla* had higher protein and ash than Berseem hay and CFM. The fatty acid profile of *Azolla* (Table 2) showed that the main saturated fatty acids (SFA) were palmitic acid (59.65%) and stearic acid (7.97%); the main monounsaturated fatty acid (MUFA) was oleic (8.35%); and the main polyunsaturated fatty acids (PUFA) were linolenic (9.45%) and linoleic (8.99%). Furthermore, the phytochemical substances of *Azolla* are presented in Table 2. *Azolla* presents values of total phenols of 5.2% of DM, total tannins of 4.31%, and total flavonoids of 0.23%. Regarding the mineral content, *Azolla* contained calcium at 0.43% of DM, magnesium at 0.59%, and iron at 0.76%.

**Table 2.** Phytochemical substances and fatty acid profile of dried *Azolla*.

Proximate composition	*Azolla pinnata*
**Total phytochemicals (% of DM)**
Total phenols	5.20
Total tannins	4.31
Total flavonoids	0.23
**Phytochemicals profile (µg/g)**
Gallic acid	300.14
Chlorogenic acid	1246.63
Methyl gallate	8.94
Caffeic acid	242.30
Ellagic acid	134.72
Vanillin	261.26
Rosmarinic acid	588.95
Resorcinol	1.00
Catechin	106.20
Rutin	162.22
Naringenin	1574.56
Daidzein	2.60
Querectin	6.97
Kaempferol	1.31
Hesperetin	7.70
Hisperidin	295.00
Apeginin	21.70
Phenantherine	12.00
Pyro catechol	23.80
Coumaric acid	23.08
Ferulic acid	1.00
Cinnamic acid	1.52
**Fatty acid profile**
Saturated fatty acids (SFA) (% of total fatty acids)
Tridecanoic acid, C13:0	1.14
Myristic acid, C14:0	0.79
Pentadecanoic acid, C15:0	0.21
Palmitic acid, C16:0	59.65
Margaric acid, C17:0	0.12
Stearic acid, C18:0	7.97
Total SFA	69.88
Monounsaturated fatty acids (MUFA) (% of total fatty acids)
Palmitoleic acid, C16:1	1.77
cis-11,14-Eicosadienoic acid, C20:1	0.09
Oleic acid, C18:1	8.35
Total MUFA	10.21
Polyunsaturated fatty acids (PUFA) (% of total fatty acids)
Linolelaidic acid, C18:2	0.75
Linoleic acid, C18:2	8.99
gamma-Linolenic acid, C18:3	0.18
Linolenic acid, C18:3	9.45
Homo-γ-linolenic acid, C20:3	0.10
Arachidonic acid, C20:4	0.46
Total PUFA	19.93

### 3.2. In vitro digestibility of nutrients, gas production, and methanogens population of rations with different levels of *Azolla*

The results revealed that *Azolla* inclusion level affected the degradability of nutrients, methane production, and the methanogens population. Group T1 showed the highest DMD and OMD, while the lowest values were observed in group T5 (p < 0.05). Groups T1 and T2 had higher NDFD, while group T4 had a lower value (p < 0.05). Furthermore, group T1 had the highest ADFD, while group T4 had the lowest value (p < 0.05) (Table 3). Moreover, the methanogen population declined with increasing *Azolla* supplementation, as the control group showed the highest population and group T5 showed the lowest population (p < 0.05) (Table 3). Furthermore, higher methane production was observed in group T3, and lower methane was observed in the control, T1, and T5 (p < 0.05) (Table 3).

**Table 3.** Effect of different levels of *Azolla* as a substitute for CFM on the means of *in vitro* DMD, OMD, NDFD, ADFD, gas production, and methanogen population.

Item	Control	T1	T2	T3	T4	T5	SEM	p-values
Degradability of nutrients								
DMD%	45.93^b^	53.13^c^	45.70^b^	44.71^b^	41.69^a^	41.42^a^	0.98	0.0001
OMD%	54.00^ab^	62.47^c^	55.47^b^	54.57^ab^	53.22^ab^	52.01^a^	0.86	0.0001
NDFD %	24.62^a^	30.79^b^	26.39^b^	25.45^a^	22.24^a^	22.69^a^	0.88	0.034
ADFD%	19.24^a^	24.72^c^	21.74^bc^	18.22^a^	17.82^a^	18.96^ab^	0.68	0.003
Gas production								
TGP, mL/0.4 g DM	98.77	100.19	92.14	99.00	95.30	95.13	0.97	0.117
CH_4_, mL/0.4 g DM	11.18^ab^	11.30^ab^	15.28^bc^	17.15^c^	13.64^c^	7.32^a^	0.94	0.012
CO_2_, mL/0.4 g DM	62.10	72.09	63.45	74.09	65.88	64.26	1.55	0.107
Total methanogens; log10 *mcrA* copies/µL								
Log 10	6.64^b^	5.91^b^	2.45^a^	2.63^a^	2.50^a^	2.17^a^	0.55	0.01

DMD = Dry matter degradability; OMD = organic matter degradability; NDFD = neutral detergent fiber degradability; ADFD = acid detergent fiber degradability; TGP = total gas production; CH_4_ = methane; CO_2_ = carbon dioxide. ^a,b,c^ Values with different superscripts within the same row differ significantly (P < 0.05).

### 3.3. Fermentation parameters of rations with different levels of *Azolla*

Higher rumen ammonia was observed in the supplemented groups (T1–T5) compared to the control group (p < 0.05) (Table 4). Group T1 showed higher numeric total VFA (p > 0.05) and higher propionic acid production (p < 0.05) (Table 4).

**Table 4.** Effect of different levels of *Azolla* as a substitute for CFM on the means of *in vitro* rumen fermentation parameters.

Item	Control	T1	T2	T3	T4	T5	SEM	p-values
pH	6.60	6.50	6.63	6.56	6.60	6.63	0.02	0.39
NH_3_-N (mg/dL)	16.22^a^	19.66^b^	23.11^b^	22.11^b^	18.72^ab^	22.44^b^	0.77	0.042
Acetic, mM	38.42	36.57	35.15	36.51	33.49	33.27	0.66	0.17
Propionic, mM	10.70^a^	13.88^b^	8.48^a^	10.84^a^	8.40^a^	8.64^a^	0.62	0.048
Isobutyric, mM	2.24	2.05	2.01	1.55	2.01	1.95	0.13	0.84
Butyric, mM	6.79	7.87	5.70	7.31	5.24	5.32	0.37	0.18
Isovaleric, mM	8.07	7.28	7.53	8.10	7.34	6.84	0.25	0.76
Valeric, mM	3.67	3.85	3.61	4.49	3.38	3.22	0.16	0.34
Total VFA, mM	69.90	71.53	62.51	68.82	59.89	59.26	1.97	0.29

VFA = Volatile fatty acids; NH_3_-N = ammonia. ^a,b,c^ Values with different superscripts within the same row differ significantly (p < 0.05).

### 3.4. Analysis of the microbial community

**Figure 1.**
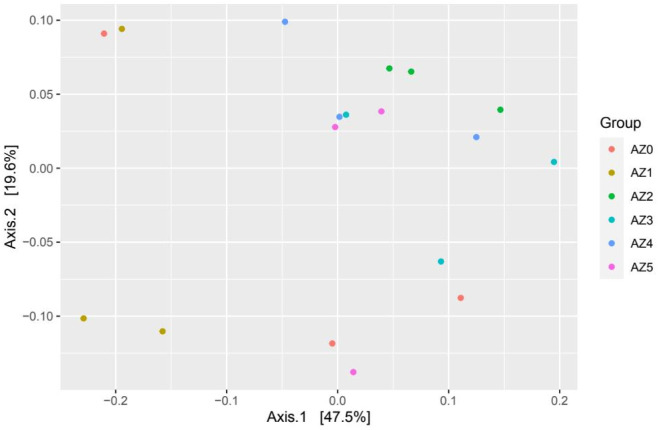
Principal coordinate analysis (PCoA) of microbial communities associated with diets supplemented with different levels of *Azolla* based on Bray-Curtis dissimilarity. The analyses were conducted between six treatments: Red circles for the control group (AZ0), brown circles for the T1 group supplemented with 10% *Azolla* of CFM (AZ1), green circles for the T2 group supplemented with 20% *Azolla* of CFM (AZ2), light blue for the T3 group supplemented with 30% *Azolla* of CFM (AZ3), dark blue for the T4 group supplemented with 40% *Azolla* of CFM (AZ4), and pink circles for the T5 group supplemented with 50% *Azolla* of CFM (AZ5).

**Diversity of the microbial community:** The Illumina amplicon sequencing of 16S rDNA genes resulted in 2,744,501 high-quality sequences with an average of 152,472 reads per sample. The rarefaction analysis revealed that the number of sequence reads was sufficient to capture all microbial species within the sample (Figure S1). *Azolla* level affected the diversity of the microbial community. A higher number of Amplicon Sequence Variants (ASVs) and Chao1 index were found in groups T2, T3, T4, and T5, while control and T1 groups revealed the lower values (p < 0.05) (Table 5; Figure S2). Furthermore, Shannon and Inverse Simpson were similar between experimental groups (p > 0.05) (Table 5). Beta diversity of the bacterial community was determined and visualized using principal coordinate analysis (PCoA) based on Bray-Curtis dissimilarity (Figure 1). The plot revealed that samples of group T1 (AZ1) and T2 (AZ2) were separated from other groups.

**Table 5.** Effect of *Azolla* level on the means of alpha diversity metrics of rumen microbial community.

	Control	T1	T2	T3	T4	T5	SEM	p-value
Observed ASVs	1150.33^a^	965.00^a^	1290.00^b^	1298.00^b^	1229.66^b^	1232.00^b^	34.62	0.02
Chao1	1150.33^a^	965.00^a^	1290.00^b^	1298.00^b^	1229.66^b^	1232.00^b^	34.62	0.02
Shannon	5.40	5.27	5.36	5.32	5.36	5.36	0.54	0.52
Invers Simpson	0.76	0.76	0.74	0.74	0.75	0.75	0.003	0.12

ASV = Amplicon Sequence Variants; ^a,b,c^ Values with different superscripts within the same row differ significantly (p < 0.05).

**Composition of bacterial community:** The bacterial community was classified into 14 bacterial phyla and one archaeal phylum, Euryarchaeota, that was affected by the *Azolla* level (p < 0.05) (Table 6). Bacterial phyla that represented more than 1% of the bacterial community were Bacteroidota (60.96%), Firmicutes (21.99%), Proteobacteria (14.39%), and Spirochaetota (1.10%). Bacterial phyla that represented less than 1% of the bacterial community were Actinobacteriota, Anaerolineae, Cyanobacteria, Desulfobacterota, Elusimicrobiota, Fibrobacterota, Fusobacteriota, Planctomycetota, Synergistota, and Verrucomicrobiota (Table 6; Figure S3).

The level of Azolla affected the relative abundance of seven bacterial phyla, including Bacteroidota, Firmicutes, Anaerolineae, Fusobacteriota, Planctomycetota, and Verrucomicrobiota (P < 0.05) (Table 6). Phylum Bacteroidota dominated the bacterial community and showed its highest relative abundance in group T1 compared to other groups (P < 0.05) (Table 6). This phylum was dominated by the families Prevotellaceae, Rikenellaceae, F082, Bacteroidales RF16 group, Bacteroidales BS11 gut group, and Muribaculaceae (Table 7). On the genus level, phylum Bacteroidota was affiliated mainly with the genus Prevotella, Prevotellaceae UCG-001, Prevotellaceae UCG-003, and Rikenellaceae RC9 gut group, which were higher in group T1 compared to other groups (P < 0.05) (Table 7).

Phylum Firmicutes was the second dominant phylum and showed its lowest relative abundance in group T1 compared to other groups (p < 0.05) (Table 6). This phylum was dominated by the families Planococcaceae, Streptococcaceae, Lachnospiraceae, Christensenellaceae, and Oscillospiraceae (Table 7). On the genus level, phylum Firmicutes was classified mainly into the genus Streptococcus, Christensenellaceae R-7 group, and Succiniclasticum, which revealed lower prevalence in group T1 compared to other groups (P < 0.05) (Table 7).

Phylum Proteobacteria represented 14.39 % of the bacterial community and was classified mainly into family Moraxellaceae, Enterobacteriaceae, Succinivibrionaceae, and Pseudomonadaceae (Table 7). On the genus level, this phylum was dominated by the genus Acinetobacter and Escherichia-Shigella. Escherichia-Shigella was higher in the control and lower in the T2 group (Table 7). Rumen methanogens belonged to phylum Euryarchaeota (Table 6), which was affiliated mainly with genus Methanobrevibacter, which had a lower prevalence in group T1 compared to other treatments (P < 0.05).

**Table 6.** Effect of *Azolla* level on the means of relative abundances (%) of bacterial and archaeal phyla.

	Control	T1	T2	T3	T4	T5	SEM	p-value
Actinobacteriota	0.03^ab^	0.02^a^	0.04^b^	0.04^ab^	0.03^ab^	0.03^ab^	0.002	0.14
Bacteroidota	62.40^a^	71.43^b^	56.27^a^	58.81^a^	57.30^a^	59.57^a^	1.42	0.003
Anaerolineae	0.08^ab^	0.052^a^	0.10^b^	0.12^b^	0.12^b^	0.09^b^	0.007	0.01
Cyanobacteria	0.17	0.18	0.15	0.17	0.14	0.19	0.03	0.48
Desulfobacterota	0.05	0.05	0.05	0.06	0.06	0.06	0.001	0.2
Elusimicrobiota	0.03	0.02	0.02	0.03	0.02	0.03	0.001	0.38
Fibrobacterota	0.10	0.09	0.08	0.06	0.05	0.08	0.007	0.13
Firmicutes	20.13^b^	13.01^a^	27.14^c^	24.00^bc^	25.62^bc^	22.06^bc^	1.27	0.002
Fusobacteriota	0.16^ab^	0.01^a^	0.54^c^	0.24^ab^	0.32^bc^	0.22^ab^	0.05	0.02
Planctomycetota	0.19^b^	0.12^a^	0.23^bc^	0.22^bc^	0.21^bc^	0.25^c^	0.01	0.001
Proteobacteria	14.90	13.21	13.86	14.44	14.39	15.52	0.33	0.48
Spirochaetota	1.09	1.08	0.97	1.18	1.08	1.20	0.02	0.07
Synergistota	0.18	0.18	0.18	0.23	0.23	0.20	0.006	0.06
Verrucomicrobiota	0.38^bc^	0.45^c^	0.26^a^	0.31^ab^	0.32^ab^	0.37^bc^	0.02	0.02
Euryarchaeota	0.05^b^	0.05^a^	0.05^ab^	0.06^abc^	0.06^bc^	0.08^c^	0.003	0.01

^a,b,c^ Values with different superscripts within the same row differ significantly (p < 0.05).

**Table 7.** Effect of *Azolla* level on the means of relative abundances (%) of dominant bacterial families and genera.

	Control	T1	T2	T3	T4	T5	SEM	p-value
P: Actinobacteriota, F: Bifidobacteriaceae								
G: *Bifidobacterium*	0.024	0.018	0.018	0.019	0.020	0.020	0.001	0.62
P: Bacteroidota
F: Prevotellaceae	32.39^b^	37.75^c^	28.61^a^	30.68^ab^	27.88^a^	30.03^ab^	0.87	0.001
G: *Prevotella*	25.00^b^	29.33	22.06^ab^	23.68^ab^	21.46^a^	23.02^ab^	0.71	0.001
G: *Prevotellaceae UCG-001*	2.39	2.42	2.24	2.23	2.31	2.49	0.03	0.24
G: *Prevotellaceae UCG-003*	1.58^bc^	1.70^c^	1.26^a^	1.42^abc^	1.16^a^	1.33^ab^	0.05	0.014
G: *Prevotellaceae YAB2003 group*	0.51^a^	1.10^b^	0.32^a^	0.54^a^	0.26^a^	0.34^a^	0.07	0.001
F: Rikenellaceae	6.62^bc^	7.05^c^	5.68^a^	6.17^ab^	6.05^ab^	6.17^ab^	0.12	0.002
G: *Rikenellaceae RC9 gut group*	6.27^bc^	6.66^c^	5.32^a^	5.78^ab^	5.67^a^	5.78^ab^	0.12	0.002
F: F082	11.20	14.56	9.95	10.33	11.54	11.44	0.48	0.05
F: Bacteroidales RF16 group	4.08	4.20	2.97	3.34	2.83	3.22	0.17	0.08
F: Bacteroidales BS11 gut group	2.43	2.57	2.60	2.50	2.53	2.30	0.04	0.19
F: Muribaculaceae	2.34	2.69	2.29	2.60	2.85	3.10	0.10	0.22
P: Firmicutes
F: Planococcaceae	1.61^a^	0.44^a^	3.53^b^	0.61^a^	3.34^b^	1.00^a^	0.35	0.003
F: Streptococcaceae G: *Streptococcus*	4.47^a^	1.20^a^	9.18^b^	9.43^b^	8.38^b^	6.40^a^	0.90	0.02
F: Lachnospiraceae	2.80	2.53	2.98	2.95	3.00	2.77	0.06	0.22
G: *Butyrivibrio*	0.21	0.17	0.22	0.21	0.24	0.20	0.007	0.19
G: *Oribacterium*	0.12^a^	0.16^a^	0.14^a^	0.14^a^	0.21^b^	0.15^a^	0.008	0.03
G: *Lachnobacterium*	0.05^c^	0.06^d^	0.04^ab^	0.04^abc^	0.04^bc^	0.04^a^	0.002	0.001
F: Christensenellaceae	1.29	0.98	1.39	1.31	1.40	1.36	0.05	0.05
G: *Christensenellaceae R-7 group*	1.16^b^	0.89^a^	1.27^b^	1.20^b^	1.29^b^	1.24^b^	0.04	0.03
F: Ruminococcaceae	0.66	0.48	0.69	0.57	0.53	0.59	0.02	0.07
F: Bacillaceae	0.42^b^	0.02^a^	0.02^a^	0.70^b^	0.05^a^	1.28^b^	0.13	0.01
F: Oscillospiraceae	2.46^b^	1.83^a^	2.58^b^	2.22^b^	2.38^b^	2.27^b^	0.06	0.001
F: Oscillospiraceae, G: *Papillibacter*	0.05^ab^	0.07^abc^	0.05^a^	0.07^abc^	0.07^bc^	0.08^c^	0.003	0.04
F: Acidaminococcaceae, G: *Succiniclasticum*	0.27^ab^	0.19^a^	0.38^b^	0.33^ab^	0.42^b^	0.40^b^	0.02	0.03
P: Fusobacteriota
F: Fusobacteriaceae, G: *Fusobacterium*	0.15^ab^	0.01^a^	0.50^c^	0.22^ab^	0.30^bc^	0.20^ab^	0.04	0.01
P: Planctomycetota
F: Pirellulaceae	0.19^b^	0.12^a^	0.23^bc^	0.22^bc^	0.21^bc^	0.25^c^	0.01	0.001
P: Proteobacteria
F: Succinivibrionaceae	1.60	1.94	1.55	1.65	1.51	1.67	0.04	0.16
F: Pseudomonadaceae	0.75^b^	0.13^a^	0.36^ab^	0.08^a^	0.33^ab^	0.09^a^	0.07	0.04
F: Moraxellaceae, G: *Acinetobacter*	7.38	8.60	7.85	8.57	8.38	9.70	0.36	0.61
F: Enterobacteriaceae, G: *Escherichia-Shigella*	3.09^c^	1.31^a^	1.77^ab^	1.54^ab^	1.96^b^	2.04^b^	0.15	0.0001
P: Spirochaetota, F: Spirochaetaceae
G: *Sphaerochaeta*	0.43^ab^	0.48^b^	0.36^a^	0.47^ab^	0.50^b^	0.56	0.019	0.03
G: *Treponema*	0.55^bc^	0.47^a^	0.51^abc^	0.58^c^	0.46^a^	0.48^ab^	0.012	0.01
P: Synergistota, F: Synergistaceae
G: *Pyramidobacter*	0.09	0.10	0.08	0.12	0.11	0.10	0.004	0.14

P = phylum; F = family; G = genus; ^a,b,c^ Values with different superscripts within the same row differ significantly (p < 0.05).

### 3.5. Principal component analysis (PCA)

**Figure 2.**
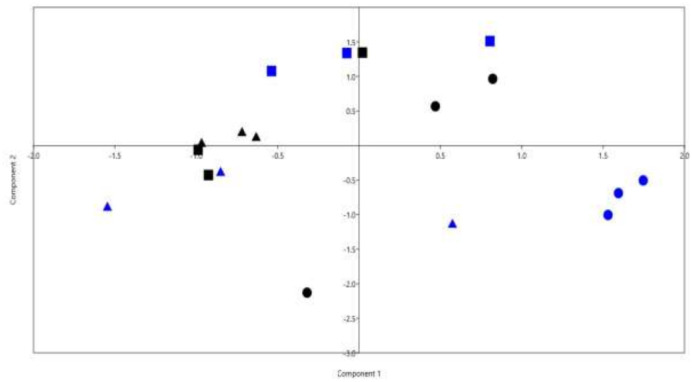
Principal component analysis (PCA) was determined using the degradability of nutrients, rumen fermentation parameters, gas production, methanogens population, and the relative abundances of dominant bacterial phyla, families, and genera. Black dots for control group, blue dots for T1 group supplemented with 10% *Azolla* of CFM, black squares for T2 supplemented with 20% *Azolla* of CFM, blue squares for T3 supplemented with 30% *Azolla* of CFM, black triangles for T4 group supplemented with 40% *Azolla* of CFM, and blue triangles for T5 group supplemented with 50% *Azolla* of CFM.

PCA analysis was performed using data on nutrients' degradability, gas production, total VFA, VFA profile, ammonia, methanogens population, and relative abundance of dominant bacterial phyla and genera (Figure 2). The results revealed that samples were separated based on the treatments. The separation was driven by total VFA, CH_4_, DMD%, and relative abundances of phylum Bacteroidota and Firmicutes.

### 3.6. Correlation analysis

**Figure 3.**
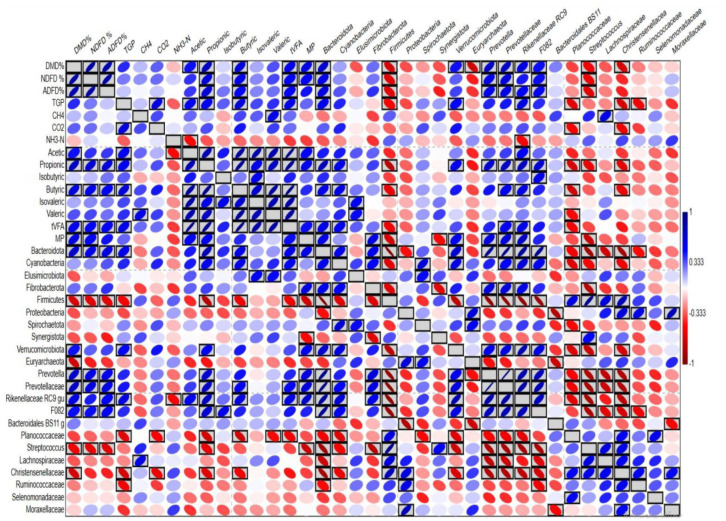
Pearson correlation analysis visualized as a heatmap. Correlation relationships were conducted between the degradability of nutrients, rumen fermentation parameters, gas production, methanogens population (MP), and relative abundances of dominant bacterial phyla, families, and genera. The black boxed ellipses refer to significant correlations at p < 0.05.

Pearson correlation analysis was conducted between nutrients' degradability, gas production, total VFA, VFA profile, ammonia, methanogens population (MP), and relative abundance of dominant bacterial phyla and genera. The correlation relationships are visualized in the heatmap (Figure 3). The heatmap revealed several positive and negative correlation relationships. For instance, there is a positive correlation between the degradability of nutrients (DMD, OMD, NDFD, and ADFD) on one side and acetic, propionic, butyric, total VFA, methanogens population (MP), and relative abundances of Bacteroidota, *Prevotella*, Prevotellaceae, and Rikenellaceae on the other side. Additionally, there is a negative correlation between the degradability of nutrients and the relative abundances of Firmicutes, *Streptococcus*, and Christensenellaceae.

## 4. Discussion

Azolla (*Azolla pinnata*) is a rich source of several nutrients and bioactive compounds that have positive effects on rumen fermentation and animal performance; therefore, it could partially substitute concentrate feed mixture for feeding ruminants. The nutritive value of *Azolla* varies according to species, season, growing conditions, climate conditions, and management system [22],[23]. Nutrient contents (OM, CP, EE, NDF, ADF, ash) of *Azolla* in the current study (Table 1) are in the range indicated in previous studies [3],[24]. Calcium content is similar to the value obtained by Parashuramulu, et al. [25].

Total phenols and tannins (5.2% and 4.3% of DM, respectively) (Table 2) are similar to the range of total phenols and condensed tannins obtained by Tran, et al. [26]. On the other hand, Kösesakal and Yıldız [24] reported higher contents of total phenols, tannins, and flavonoids than the values indicated in the current study. Moreover, the fatty acid profile (Table 2) was similar to previous studies [27],[28]. Thus, incorporating *Azolla* into animal rations at different levels would lead to variation in the chemical composition of animal rations. The composition of the diet is the main driver of the rumen microbial community and diet fermentation in the rumen [7].

### 4.1. Nutrient degradability and fermentation

In this study, dietary *Azolla* level affected the degradability of nutrients (DMD, OMD, NDFD, and ADFD) and the production of gases and VFAs (Table 3), which agrees with previous studies [9],[25]. Parashuramulu, et al. [25] reported that the DMD and OMD for *Azolla* were 79.5% and 63.8%, higher than the degradability of *Azolla*-based rations in the current study. The highest degradability of nutrients was obtained when *Azolla* was incorporated at 10%–20 % of the concentrate mixture (Table 3), which agrees with a previous *in vitro* study [9]. On the other hand, increasing *Azolla*'s level decreased the degradability indices. Similarly, the digestibility of nutrients declined in lambs supplemented with 20% *Azolla* [4].

The decline in digestibility with increasing *Azolla* level could be attributed to the decline in available energy and increasing phenolic substances with antimicrobial activities, which affect the rumen microbial community [7],[9]. Furthermore, increasing dietary *Azolla* increases ash intake, which affects digestibility negatively [10]. The current results of the *in vitro* experiment were supported by some *in vivo* studies on goats and sheep, which recommended 10%–20% as suitable *Azolla* inclusion levels [1],[3],[5]. Higher VFA and propionic acid production were observed in the T1 group supplemented with 10% *Azolla* of CFM (Table 4). Similarly, lactating goats supplemented with 10% *Azolla* showed higher digestibility and VFA production [3]. Furthermore, higher VFA production was observed in growing goats supplemented with phytogenic mixture at 1% of DM intake [7] and buffalo supplemented with flavonoids [29], which indicated that a moderate level of phytogenic substances guarantees positive effects on rumen fermentation and animal performance.

### 4.2. Methane production and the methanogen population

The decline in rumen methanogens and methane production in group T5 (50% *Azolla*) could be explained by the lower availability of growth substrate due to lower digestibility, as well as the increases in phytogenic substances that impact rumen methanogens negatively [7]. Lower methane production in group T1 was associated with higher propionic acid production (Table 4). Propionic production consumes hydrogen from the rumen, which decreases methane production as hydrogen is the main substrate in methane production [3],[7].

### 4.3. Shifts in microbial community

Variations in the dietary degradability and fermentation parameters were associated with shifts in the microbial communities. To the best of our knowledge, this is the first study that investigated the rumen microbial communities associated with diet, including dried *Azolla*. Group T1 showed lower microbial diversity compared to other groups, which agrees with findings on sheep supplemented with microalgae [30] (Table 5).

Phylum Bacteroidota and Firmicutes dominated the bacterial community in the current study (Table 6), which agrees with previous studies that used microalgae and phytochemicals [7],[30]. The relative abundance of phylum Bacteroidota was enhanced, and the relative abundance of Firmicutes declined in T1 compared with the control diet and other treatments. A similar trend was obtained in goats supplemented with phytochemical substances [7] and sheep supplemented with microalgae [30]. Genus *Prevotella* dominated the bacterial communities in the current study (Table 7) and the microbiomes of other ruminants; this genus is a key player in rumen fermentation because it degrades a wide range of substrates such as protein, peptides, hemicellulose, and protein and produces propionic acid [30]–[32]. This genus was enriched in group T1 (10% *Azolla*), which explains the higher digestibility and propionic acid with low methane production in this group. Higher *Prevotella* in group T1 could be attributed to the availability of growth substrates [7],[30],[31]. In the same line, T1 treatment showed higher relative abundances of *Rikenellaceae RC9 gut group* and Family F082, which have important roles in the digestion of dietary fiber [7],[33]. Family Bacteroidales RF16 group was noted in the rumen of Yak fed a high-forage diet [34]. Family Bacteroidales BS11 gut group has an important role in the degradation of hemicellulose and was enriched in the rumen of Alaskan moose fed high-forage diets [35]. These findings, along with the current results, indicate that the T1 diet supports the growth of bacterial groups specialized in the fermentation of structural polysaccharides, which was reflected in higher degradability and VFA production.

The phylum Firmicutes was classified mainly to the genus *Streptococcus*, which declined in T1 compared with other treatments. This genus produces lactic acid when the rumen pH is lower than 5.5, which increases the rumen acidosis [36], which is a positive point for the inclusion of *Azolla* in animal diet. Candidate genus *Christensenellaceae R-7 group* has an important role in dietary fiber degradation and animal feed efficiency [37],[38]. This genus was higher in *Azolla*-supplemented rations except for T1. The phylum Proteobacteria was dominated by *Escherichia-Shigella*, which has a pathogenic effect and causes diarrhea [39]. The decline of this genus due to *Azolla* supplementation highlights its potential positive effects on animal performance.

Rumen methanogens were affiliated with the phylum Euryarchaeota and genus *Methanobrevibacter*, which declined in T1 compared to other treatments (p < 0.05). *Methanobrevibacter* is the main methane producer, and it produces methane using hydrogen, acetate, and formate, which leads to energy losses [7]. Additionally, this genus was associated with low feed efficiency in steers [40]. The decline of *Methanobrevibacter* in *Azolla*-supplemented groups could be a result of the presence of phenols and tannin compounds that have a direct negative impact on rumen methanogens [7]. Furthermore, polyunsaturated fatty acids in the diet reduce the rumen methanogens by decreasing the available hydrogen for methane production [30],[41]. Additionally, phytogenic substances decrease rumen protozoa, which provide methanogens with hydrogen through fiber degradation [7],[41]. In the current study, the digestibility of NDF and ADF declined with increasing *Azolla* supplementation in groups T3, T4, and T5.

These findings explain that *Azolla* supplementation modified the rumen microbial community, improved the degradability of nutrients, improved VFA production, and decreased methane production, which could have a positive impact on animal performance and the environment.

## 5. Conclusions

*Azolla* is a rich source of several nutrients and phytochemicals, which modify the rumen microbial community and affect rumen fermentation, methane production, and the degradability of nutrients. The inclusion of *Azolla* at 10%–20% of the concentrate feed mixture encouraged the fiber-degrading bacteria and declined rumen methanogens, which improved the degradability of nutrients and VFA production and decreased methane production. Therefore, it is recommended to use these levels in future *in vivo* studies to assign the best inclusion level in animals' diets based on the results of animal performance, rumen microbiota, rumen fermentation, methane production, and nutrient digestibility.

## Use of AI tools declaration

The authors declare they have not used Artificial Intelligence (AI) tools in the creation of this article.


