# Supplementary material for: Degradability, rumen fermentation, and rumen microbiota of livestock rations containing different levels of Azolla pinnata
Source: AIMS Microbiol. 2025 Aug 1;11(3):679–98. doi: 10.3934/microbiol.2025028 (PMC12511962; doi:10.3934/microbiol.2025028)
Supplement: Supplementary file 1 [file microbiol-11-03-028-s001.pdf]

AIMS Microbiology

AIMS Microbiology, 11(3): 679–698.
DOI: 10.3934/microbiol.2025028
Received: 19 February 2025
Revised: 24 June 2025
Accepted: 18 July 2025
Published: 01 August 2025

https://www.aimspress.com/journal/microbiology

Research article

Degradability, rumen fermentation, and rumen microbiota of livestock rations containing different levels of *Azolla pinnata*

Ahmed. F.A. Abd-Elgwad[1], Salah Abo Bakr[1], Ebrahim A. Sabra[2], Eman A. Elwakeel[3], Mahmoud. M. Khorshed[4], Hamdy. M. Metwally[4] and Alaa Emara Rabee[1,*]

[1] Animal and Poultry Nutrition Department, Desert Research Center, Cairo, Egypt
[2] Genetic Engineering and Biotechnology Research Institute, University of Sadat City, Sadat City, Egypt
[3] Animal and Fish Production Department, Faculty of Agriculture, Alexandria University, Alexandria
[4] Animal Production Department, Faculty of Agriculture, Ain-Shams University, Cairo, Egypt

* **Correspondence:** Email: Rabee_a_m@yahoo.com; Tel: +201096884139.

Supplementary

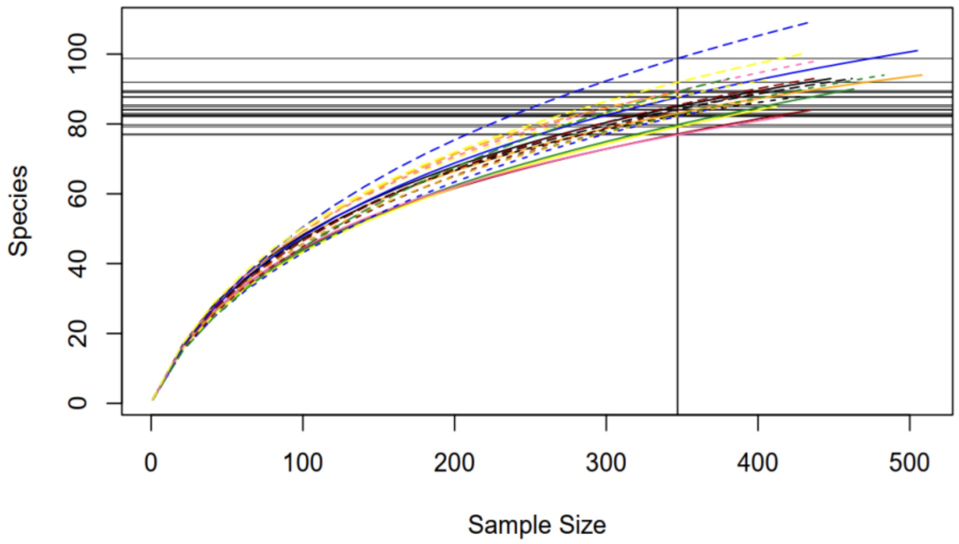

Figure S1. Rarefaction analysis of microbial communities associated with rations with different levels of *azolla*.

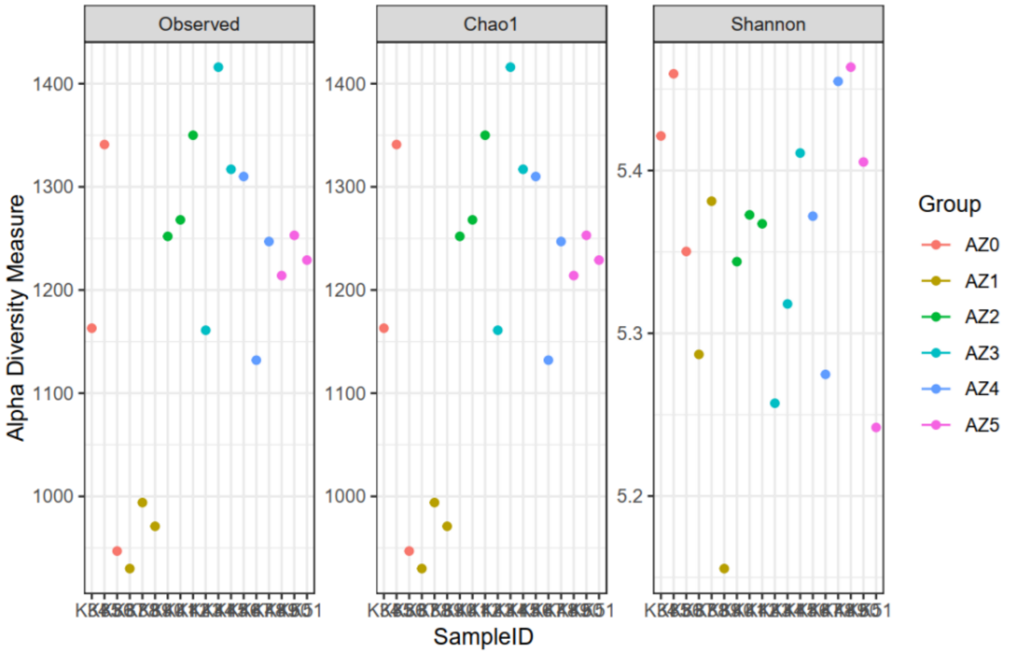

Figure S2. Alpha diversity indices of microbial communities associated with ration supplemented with different levels of *azolla*. Control group (AZ0), T1 group supplemented with 10% *azolla* of CFM (AZ1), T2 group supplemented with 20% *azolla* of CFM (AZ2), T3 group supplemented with 30% *azolla* of CFM (AZ3), T4 group supplemented with 40% *azolla* of CFM (AZ4), and T5 group supplemented with 50% *azolla* of CFM (AZ5).

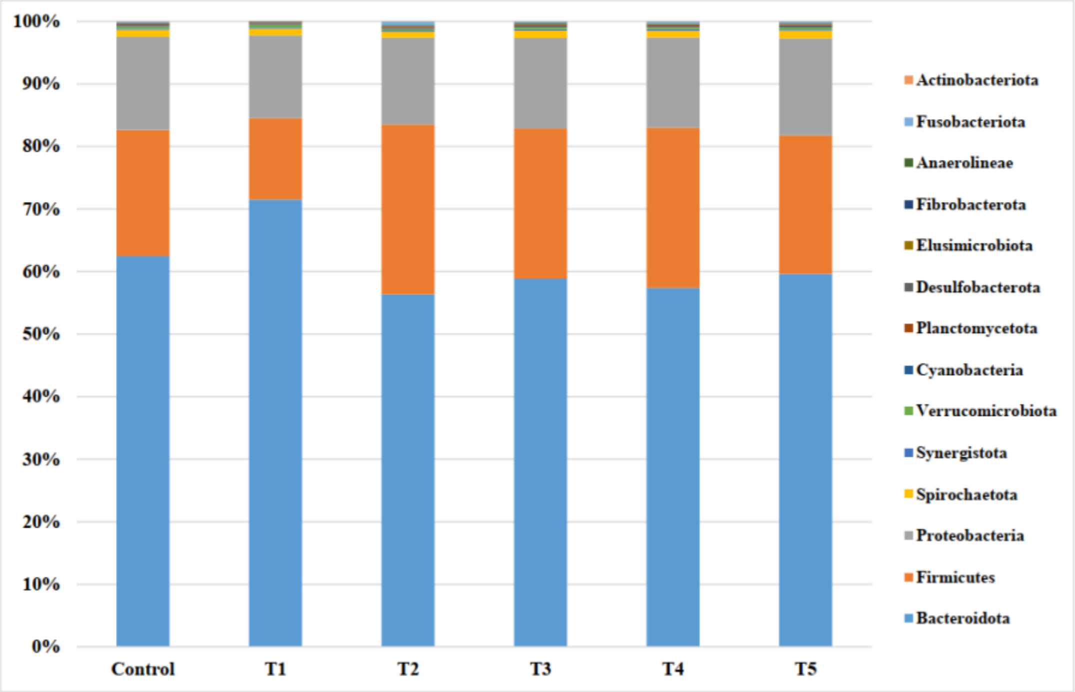

Figure S3. The relative abundances (%) of bacterial phyla associated with the ration supplemented with different levels of *azolla*. Non-supplemented ration (Control), T1 group supplemented with 10% *azolla* of CFM, T2 group supplemented with 20% *azolla* of CFM, T3 group supplemented with 30% *azolla* of CFM, T4 group supplemented with 40% *azolla* of CFM, and T5 group supplemented with 50% *azolla* of CMF.

AIMS Press

© 2025 the Author(s), licensee AIMS Press. This is an open access article distributed under the terms of the Creative Commons Attribution License (https://creativecommons.org/licenses/by/4.0)
